# A Virus Infecting Marine Photoheterotrophic Alphaproteobacteria (*Citromicrobium* spp.) Defines a New Lineage of ssDNA Viruses

**DOI:** 10.3389/fmicb.2018.01418

**Published:** 2018-06-27

**Authors:** Qiang Zheng, Qi Chen, Yongle Xu, Curtis A. Suttle, Nianzhi Jiao

**Affiliations:** ^1^State Key Laboratory of Marine Environmental Science, Institute of Marine Microbes and Ecospheres, Xiamen University, Xiamen, China; ^2^College of Ocean and Earth Sciences, Xiamen University, Xiamen, China; ^3^Institute of Marine Science and Technology, Shandong University, Jinan, China; ^4^Departments of Earth, Ocean and Atmospheric Sciences, Microbiology and Immunology, Botany, The Institute for the Oceans and Fisheries, The University of British Columbia, Vancouver, BC, Canada

**Keywords:** *Microviridae*, *Citromicrobium*, ssDNA phage, ssDNA prophage, photoheterotrophic bacteria

## Abstract

In recent metagenomic studies, single-stranded DNA (ssDNA) viruses that infect bacteria have been shown to be diverse and prevalent in the ocean; however, there are few isolates of marine ssDNA phages. Here, we report on a cultivated ssDNA phage (vB_Cib_ssDNA_P1) that infects *Citromicrobium bathyomarinum* RCC1878 (family *Sphingomonadaceae*), and other members of the genus. This is the first ssDNA phage reported to infect marine alphaproteobacteria, and represents a newly recognized lineage of the *Microviridae* infecting members of *Sphingomonadaceae*, the *Amoyvirinae*. The ∼26 nm diameter polyhedral capsid contains a 4,360 bp genome with 6 open reading frames (ORFs) and a 59.3% G+C content. ORF1 encodes the capsid protein and ORF3 encodes the replication initiator protein. The replication cycle is ∼5 h, followed by a burst releasing about 180 infectious particles. The closest relative of vB_Cib_ssDNA_P1 is a prophage within the genome of *Novosphingobium tardaugens* strain NBRC16725. Phylogenetic analysis indicates that the vB_Cib_ssDNA_P1 phage and two related prophages, as well as an environmental sequence, form a novel group within the *Microviridae.* Our results indicate that this is a previously unknown lineage of ssDNA viruses which also supplies a new model system for studying interactions between ssDNA phages and marine bacteria.

## Introduction

Viruses are the most abundant biological entities in the ocean and can influence microbial population dynamics through infection and cell lysis (Fuhrman, 1999; Weinbauer and Rassoulzadegan, 2004; Suttle, 2005), with consequent effects on nutrient cycling (Wommack and Colwell, 2000; Suttle, 2007). Most characterized viruses that infect marine bacteria, by far, are double-stranded DNA (dsDNA) phages. However, recent metagenomic studies demonstrate that single-stranded DNA (ssDNA) phages, such as those in the family *Microviridae*, are prevalent in diverse environments (Angly et al., 2006; Labonté and Suttle, 2013a; Székely and Breitbart, 2016). The abundances of ssDNA phages in *in situ* environments are underestimated because of the weak staining capacities with the small size and the single-stranded nature of their genomes (Holmfeldt et al., 2012; Brum et al., 2013). Nevertheless, the recent quantitative viromics suggested that ssDNA phages might be a minor fraction of DNA viral communities (Roux et al., 2016).

Members of the *Microviridae* infect bacteria, and have ∼4.5–6.1 kB positive-sense circular ssDNA genomes enclosed within small (∼30 nm) icosahedral capsids (Fane et al., 2006). The DNA is replicated using a rolling-circle mechanism, and encodes two relatively conserved genes for a capsid and replication initiator (Brentlinger et al., 2002; Labonté and Suttle, 2013b). Members of the *Microviridae* are usually classified into either the subfamily *Bullavirinae* (formally *Microviridae* including genus *Microvirus*) or *Gokushovirinae*, based on the structure of the viral particle and composition of the genome (Brentlinger et al., 2002; Krupovic et al., 2016). *Gokushovirinae* have genome ranging from 4.4 to 4.9 kB including nine genes, whereas *Bullavirinae* contain 11 genes with genome size from 5.4 to 6 kB (Fane et al., 2006; Pearson et al., 2016). Previous studies indicated that members of this family have a limited host-range. Viruses in the genus *Microvirus* infect cells in the family *Enterobacteriaceae* (e.g., *Escherichia coli*) (Chipman et al., 1998; Brentlinger et al., 2002) and are typified by the microvirus phiX174. In contrast, members of the subfamily *Gokushovirinae* are specialized to infect intracellular parasites, such as *Chlamydia*, *Bdellovibrio*, and *Spiroplasma* (Chipman et al., 1998; Brentlinger et al., 2002; Everson et al., 2002; Garner et al., 2004), although recent isolation-independent investigations have revealed gokushoviruses associated with *Gammaproteobacteria* (Roux et al., 2012a). In addition, *Microviridae*-like genomes have been discovered in the genomes of bacteria within the *Bacteroidetes* (Krupovic and Forterre, 2011), suggesting a lysogenic lifestyle.

Phages belonging to the *Microviridae* were once thought to have a limited distribution; however, metagenomic studies indicate they are likely widespread across diverse habitats. For example, viruses belonging to the *Microviridae* have been detected in seawater (Angly et al., 2006; Tucker et al., 2011; Holmfeldt et al., 2012; Labonté and Suttle, 2013b), methane seeps (Bryson et al., 2015), freshwater (López-Bueno et al., 2009; Roux et al., 2012a,b; Zhong et al., 2015), Sphagnum-dominated peatland (Quaiser et al., 2015), sewage and sediment (Hopkins et al., 2014), the human gut and feces (Roux et al., 2012b), and stromatolites (Desnues et al., 2008). Only eight ssDNA phages have been isolated for marine heterotrophic bacteria (Holmfeldt et al., 2012), and all infect *Cellulophaga* spp. (*Bacteroidetes*). In addition, a microvirus-like phage has been induced from a marine *Synechococcus* isolate (McDaniel et al., 2006). Therefore, although there is a broad distribution of ssDNA viruses in the sea, there are few representative isolates.

Members of the genus *Citromicrobium* are marine aerobic anoxygenic photoheterotrophs (AAPs) in the family *Sphingomonadaceae* and the class *Alphaproteobacteria* (Yurkov et al., 1999; Zheng et al., 2016). *Citromicrobium*-like AAPs mainly occur in the oligotrophic ocean and are more abundant in the upper twilight zone (150–200 m depth) than in subsurface waters (5 and 25 m) of the western Pacific Ocean (Zheng et al., 2015). Prophage are widely present in *Citromicrobium* strains, as revealed by a comparison of eight Citromicrobial genomes that share identical 16S rRNA sequences but were isolated from diverse environments (Zheng et al., 2014, 2016). Despite the importance of viruses as agents of mortality, and in nutrient cycling in the oceans (Fuhrman, 1999; Suttle, 2005, 2007), there are still relatively few model systems for viruses infecting marine bacteria; this is especially true for photoheterotrophs. Moreover, the limited number of viruses that have been isolated, which infect marine photoheterotrophs (members of genera *Roseobacter* and *Dinoroseobacter*), have been tailed phages belonging to the order *Caudovirales* (Zhang and Jiao, 2009; Ji et al., 2015). Here, we report the first ssDNA phage found to infect marine alphaproteobacterial photoheterotrophs (*Citromicrobium* spp.). It represents a previously unrecognized lineage of ssDNA viruses (*Amoyvirinae*) that infect members of the *Sphingomonadaceae*.

## Materials and Methods

### Bacterial Strains

*Citromicrobium bathyomarinum* RCC1878, an isolate from the Mediterranean Sea, was provided by Dr. Christian Jeanthon at the Roscoff Culture Collection, and used as a host for phage isolation. As well, the following 13 alphaproteobacterial strains were used to test host-range. Four of them were purchased from the Deutsche Sammlung von Mikroorganismen und Zellkulturen: *Dinoroseobacter shibae* DLF 12, *Roseobacter denitrificans* DSM 7001, *Erythrobacter litoralis* DSM 6997, and *Erythrobacter longus* DSM 8509, as well, *Roseibacterium beibuensis* JLT1202, *Roseibium* sp. 1Q1, *Porphyrobacter* sp. YT40, and six strains of *Citromicrobium* spp. (JL31, JL89-1, JL477, JL1010, JL1351, and JL2201) from our lab at Xiamen University. The cross-infectivity was tested by double-agar layering and spotting phage suspensions onto a bacterial lawn. All strains were grown in rich organic medium (containing 1.0 g yeast extract, 1.0 g Bacto Peptone, and 1.0 g sodium acetate per liter of artificial seawater with vitamins and trace element) (Yurkov et al., 1999) at 28°C.

### Isolation of Phages

Water samples were collected from a marine aquaculture site around Xiamen coast, China, on 24 January 2016 and immediately filtered through 0.2-μm pore-size polycarbonate membrane filters (Millipore, Bedford, MA, United States). A 100 mL of filtrate was added to 150 mL of exponentially growing bacterial culture and incubated overnight at 28°C and 160 rpm in a shaker. Cultures were then centrifuged at 10,000 ×*g* for 20 min to remove bacteria. A 100 μL subsample of cell-free supernatant was added to 1 mL of exponentially growing culture, and then plaque assayed as described by Suttle and Chen (1992). Each phage isolate was plaque purified at least three times.

### One-Step Growth Experiments

One-step growth assays of the phage isolates were performed according to Middelboe et al. (2010). Briefly, the viruses were suspended in SM buffer (10 mM NaCl, 50 mM Tris, 10 mM MgSO_4_, and 0.1% gelatin, pH 7.5) and added to triplicate exponentially growing cultures (OD = 0.3) at a multiplicity of infection (MOI) of 0.1. After 10 min, cells were removed by centrifugation, leaving the unadsorbed viruses, which were poured off with the supernatant. The bacteria in the pellet were resuspended in medium and 50 μL were transferred to 50 mL of medium and incubated at 28°C. Subsamples were taken every hour for 7 h. The number of plaque forming units (PFUs) was determined by plaque assay. The latent period was determined from the time of phage adsorption to the appearance of the first free phage in the medium during a one-step growth experiment. Concurrently, the burst size was calculated from the number of PFUs (i.e., free viruses) at the end of the first viral rise divided by the PFUs (i.e., infected cells) at the initial time.

### Phage Amplification and Purification

The phage suspension was added to 1 L of host culture at an OD_600_ of 0.2–0.3, and an MOI of 0.01, and incubated overnight at 28°C and 180 rpm in a shaker. Phage particles in lysates were harvested and purified as described by Chen et al. (2006) with some modifications. Briefly, phage lysates were treated with 2 mg DNase I and 2 mg RNase A at room temperature for 1 h. After digestion, the NaCl concentrations of the lysates were adjusted to 1M and incubated at 4°C for 1 h. The treated lysates were centrifuged at 10,000 ×*g* for 15 min in a Beckman AR centrifuge. Supernatants were collected and filtered through a 0.2-μm pore-size filter to remove the remaining cells and debris at 10,000 ×*g*, 4°C for 15 min in a Beckman AR centrifuge. The filtrate was then concentrated by polyethylene glycol 8000 precipitation. The precipitate was resuspended in 6 mL SM buffer and incubated overnight at 4°C. Subsequently, the phage suspensions were overlaid onto 1.3–1.6 w/v CsCl gradients at 0.1 intervals and centrifuged for 24 h at 200,000 ×*g* using a SW41 Ti rotor in a Beckman Coulter Optima L-100 XP Ultracentrifuge. The visible phage bands (in 1.5 w/v CsCl density) were extracted, and the CsCl in the phage suspensions was removed by centrifugal ultrafiltration.

### Transmission Electron Microscopy

One drop of the CsCl-purified phage suspension was adsorbed to a Formvar/carbon-coated 200-mesh copper grid for 10 min and negatively stained with 0.5% (wt/vol) aqueous uranyl acetate in the dark for 30 s. After drying for 2 h, the grid was examined using a JEM-2100 transmission electron microscope at 80 kV. Images were taken using the GATAN INC CCD image transmission system.

### Extraction of Phage DNA and Genome Sequencing

CsCl-purified phage were treated with a proteinase K cocktail (100 μg/mL proteinase K, 50 mM Tris, 25 mM EDTA, and 1% SDS) at 55°C for 3 h. The phage nucleic acid was extracted using the phenol/chloroform method. The ssDNA was then isopropanol-precipitated, washed with ethanol, and dissolved in TE buffer (10 mM Tris, 1 mM EDTA). Phage DNA was amplified using multiple-displacement amplification to convert ssDNA into dsDNA and then resuspended in TE buffer. To identify the nature of the phage genome, nucleic acid digestion using different enzymes was performed. Exonuclease S1 which digests ssDNA and restriction enzymes including Acc I, Alu I, Dpn I which digest dsDNA were used to determine whether the genome was single- or double-stranded. PCR method and BAL-31 nuclease digestion of the phage genome was used to testify whether the genome was linear, circular or circularly permuted (Yoshida et al., 2008).

The genome was sequenced by the Illumina MiSeq system for libraries with insert sizes of ∼395 bp. Paired-end reads of average 250-bp length were assembled using Velvet (v2.8) (Zerbino and Birney, 2008), with coverage >1,000×. Open reading frames (ORFs) were predicted using ORF Finder^1^ and GeneMarkS (Besemer and Borodovsky, 1999). Translated ORFs were compared with known protein sequences using BLAST (Altschul et al., 1990).

### Proteomics Analysis

The purified phage particles were treated with STD buffer (4% SDS, 100 mM DTT, 150 mM Tris–HCl pH 8.0) in a water bath at 99°C for 10 min. After centrifugation (16,000 ×*g*, 4°C, 30 min), the supernatants were stored at -80°C for further proteomic analysis. Protein digestion was performed according to a previously described FASP procedure (Wiśniewski et al., 2009). Proteome analysis was performed on Obitrap Fusion mass spectrometer (Thermo Finnigan, San Jose, CA, United States) that was coupled to an Easy-nLCTM 1000 (Thermo Fisher Scientific, Waltham, MA, United States). MS/MS spectra were searched using a MASCOT engine (Matrix Science, London, United Kingdom; version 2.2) against the phage genome.

### Phylogenetic Analysis

The complete major capsid amino acids sequences were used to construct a phylogenetic tree. All sequences collected from the NCBI database were aligned using Clustal X. Columns with gaps were deleted. Maximum-likelihood phylogenetic trees were constructed using RAxML 7.3.0 (Stamatakis, 2006) with the “PROTGAMMALG” model, which assumes that amino acid substitution rates among 289 sites follow a gamma distribution. The phylogenetic trees were supported by bootstrap analysis for the resampling test with 100 replicates.

### Nucleotide Sequence Accession Number

The GenBank accession number assigned to the complete vB_Cib_ssDNA_P1 is MG214764.

## Results and Discussion

In order to isolate phage of marine photoheterotrophic bacteria, we used *C. bathyomarinum* strain RCC1878 as a host to screen for viruses. Its genome contains a complete prophage, implying that it is subject to viral infection. Screening against this host resulted in the isolation of a ssDNA virus that belongs to a previously unrecognized lineage of phages (*Amoyvirinae*) that infect members of the *Sphingomonadaceae*. A detailed description of this virus is discussed in detail below.

### Morphology, Growth, and Basic Biology of vB_Cib_ssDNA_P1

**FIGURE 1:**
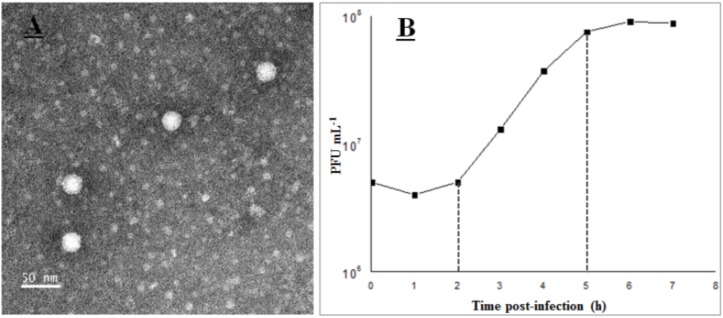
Transmission electron microscopy images **(A)** and one-step curve **(B)** of phage vB_Cib_ssDNA_P1.

Phage vB_Cib_ssDNA_P1 was isolated from surface water near a coastal aquaculture site using the double-agar layer method, and was found to produce large, clear, round plaques with regular edges. The phage has a polyhedral capsid (∼26 nm in diameter) that is significantly smaller than those of described marine dsDNA phages (podo-, sipho-, and myoviruses), and lacks a tail (**Figure 1A**). Morphologically, it was similar to members of the *Microviridae*. According to the nucleic acid digestion result, the phage DNA could be digested by S1 nuclease rather than by restriction enzymes *Acc* I, *Alu* I, and *Dpn* I. This proved its genome was single-strand nucleic acids.

The growth characteristics of vB_Cib_ssDNA_P1 were evaluated by a one-step growth on *C. bathyomarinum* RCC1878 (**Figure 1B**). The first 2 h of the ∼5 h replication cycle were classified as the early stage, when almost no viral particles were released. This stage comprises the latent phase, when phages adsorb and inject their genomes into the host cells, and biosynthesis of the viral components occurs, but phage particles are not released. In the subsequent late stage, between ∼3 and 5 h after adsorption, progeny viral particles are assembled and released. The number of released phage particles reached a plateau at 5 h after infection, with a burst size of ∼180 viral particles per infected cell.

### Genomic and Proteomic Features

To further investigate the new virus, its genome was sequenced. The genome of vB_Cib_ssDNA_P1 is 4,360 bp of circular, ssDNA, which is relatively small among the *Microviridae* (∼4.5–6.1 kB). The G+C content is 59.3%, lower than the 64.8% of its host, *C. bathyomarinum* RCC1878 (Zheng et al., 2016). The six identified ORFs in vB_Cib_ssDNA_P1, comprised 92.4% of the genome (**Table 1**).

**Table 1:** Predicted ORFs and their annotation.

ORFs	Start	Stop	Length	G+C	MW (kDa)	Function
1	31	1,317	1,287	0.595	47.54	Capsid
2	1,328	1,738	411	0.535	14.94	–
3	1,847	2,641	795	0.611	30.23	Replication initiator
4	2780	4,024	1,245	0.599	45.73	–
5	4,014	4,190	177	0.616	5.89	–
6	4,217	4,330	114	0.623	4.05	–

High-resolution LC-mass spectrometry was used to analyze proteomic characterization of virion particles. Only one protein was identified, and it is the most abundant structural protein with the molecular weight of 47.5 kDa (**Table 1** and **Supplementary Figure S1**). Furthermore, all sequenced peptides matched ORF1. So here, ORF1 was annotated as major capsid protein.

Based on similarity to other sequences in GenBank, ORF3 encodes the replication initiator protein; whereas, ORFs 2, 5, and 6 had no significant matches and a relatively wide range of G+C content from 53.5 to 62.3%. ORF4 is a conserved hypothetical gene.

**FIGURE 2:**
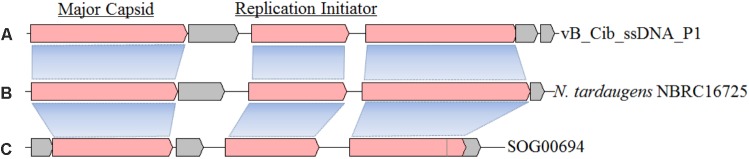
The structure and gene compositions of phage vB_Cib_ssDNA_P1 **(A)** and two other close relatives, including a prophage found in *Novosphingobium tardaugens* NBRC 16725 (accession no. BASZ01000013) **(B)** and the environmental ssDNA viral sequence SOG00694 (accession no. JX904102) **(C)**.

Similarity analysis demonstrated that the closest matches for ORFs 1, 3, and 4, are with the genome of *Novosphingobium tardaugens* strain NBRC16725, a bacterium that belongs to the same family as *C. bathyomarinum* RCC1878 (*Sphingomonadaceae*) (**Figures 2A,B**). These results suggest that there is a ssDNA prophage (∼4,207 bp with 57.9% G+C content) in the genome of *N. tardaugens* NBRC16725 (accession no.: BASZ01000013; position: from 78287 to 82493). Residual gene fragments that display significant amino-acid identities with ORFs 1 (38%, with BLASTP e-value 1e-78) and 4 (35%, 7e-11) are also found in genome of the bacterium, *Sphingomonas* sp. 67-36, which contains an incomplete ssDNA prophage (accession no.: MKSU01000016; position: from 84561 to 88136). In addition, an environmental ssDNA viral genome (SOG00694, accession no. JX904102) from the Strait of Georgia, British Columbia is 3,926 bp in length with a 58.6% G+C content, and displays ORFs similar to ORFs 1 (42%, 4e-83) and 3 (52%, 1e-55) (**Figure 2C**) (Labonté and Suttle, 2013a). ORFs 1 and 3 also have homologous genes with related ssDNA viral genomes (**Figure 2**), while ORF 4 shows gene fragment recombination. The other small genes of vB_Cib_ssDNA_P1 (ORFs 2, 5, and 6) have no homologs within the other two genomes (**Figure 2**).

### Host-Range

To assess the range of bacteria that can serve as hosts for vB_Cib_ssDNA_P1, we applied the virus to fourteen related alphaproteobacterial strains using double-agar layering and spotting phage suspensions. As shown in **Table 2**, all seven *Citromicrobium spp.* strains, which shared identical 16S rRNA sequences, were susceptible to vB_Cib_ssDNA_P1 infection; however, no close-relatives belonging to the genus *Erythrobacter* or other genera could be infected, indicating that this phage has a relatively narrow host-range. Nevertheless, only strain RCC 1878 formed large circular clear plaques (∼1 cm diameter) 24 h after infection. Plaques in the other six *Citromicrobium* strains after 24 h were relatively small (<0.3 cm diameter), suggesting that strain RCC1878 may be the preferred host.

**Table 2:** Bacterial susceptibility to phage vB_Cib_ssDNA_P1.

Genus/clade	Strain	Origin	Susceptibility
*Citromicrobium*	RCC1878	Mediterranean Sea	+
	JL477	South China Sea	+^∗^
	JL2201	South Atlantic Ocean	+^∗^
	JL1351	South China Sea	+^∗^
	JL1010	South China Sea	+^∗^
	JL89-1	South China Sea	+^∗^
	JL31	South China Sea	+^∗^
*Erythrobacter*	DSM 6997	Seaweed	–
	DSM 8509	Seaweed	–
*Porphyrobacter*	YT40	Coastal	–
*Roseobacter* clade	DLF 12^a^	Seaweed	–
	DSM 7001^b^	Seaweed	–
	JLT1202^c^	South China Sea	–
	1Q1^d^	Coastal	–

*^∗^Means relatively small size of plaques. ^a^Dinoroseobacter shibae DLF12, ^b^Roseobacter denitrificans DSM 7001, ^c^Roseibacterium beibuensis JLT1202, ^d^Roseibium sp. 1Q1.*

### Phylogenetic Analysis of *Amoyvirinae*

**FIGURE 3:**
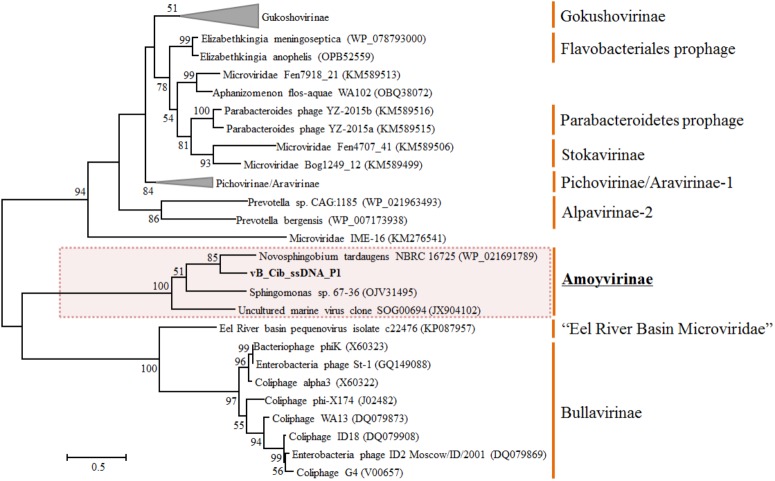
Unrooted maximum-likelihood phylogenetic tree based on the full-length major capsid inferred amino-acid sequences in *Microviridae* genomes. Bootstrap percentages from maximum-likelihood (100 replications) are shown. The scale bar represents a distance of 0.2 substitutions per site.

To explore the evolution and to classify vB_Cib_ssDNA_P1, we performed a phylogenetic analysis. Based on the phylogeny of the major capsid protein sequences, the phage vB_Cib_ssDNA_P1 and its three closest relatives form a novel group within the *Microviridae* (**Figure 3**). The host “*Citromicrobium*” belongs to the family *Sphingomonadaceae*, while the complete and incomplete related ssDNA prophages are also found in the genomes of other member of the family, *N. tardaugens* NBRC16725 and *Sphingomonas* sp. 67-36. To date, the known hosts for *Amoyvirinae* are members of the *Sphingomonadaceae*, although the host associated with the environmental metagenomic sequence is unknown. The absence of integrase-related genes in the prophage suggests that *Amoyvirinae* rely on cellular chromosome dimer resolution machinery (Krupovic and Forterre, 2011).

Based on phylogenetic analyses of the capsid and replication initiator sequences, which display the similar tree topology, *Amoyvirinae* are phylogenetically related to the Eel River Basin *Microviridae* (Bryson et al., 2015) (**Figure 3** and **Supplementary Figure S2**). Our data are consistent with *Amoyvirinae* being a divergent lineage within the *Microviridae*.

Aerobic anoxygenic photoheterotrophs, as an important functional group, comprise up to 20% of total bacterioplankton in the coastal oceans, and play significant roles in marine carbon and energy cycling (Jiao et al., 2010; Koblížek, 2015). However, only limited number of phages infecting AAPs have been isolated to date, and they are all tailed phages mainly infecting members of genera *Roseobacter*, *Dinoroseobacter*, and *Erythrobacter* (Zhang and Jiao, 2009; Ji et al., 2015; Lu et al., 2017). In addition, an inducible sipho-like bacteriophage was isolated from *Citromicrobium* sp. JL354 (Zheng et al., 2014). Here, the ssDNA phage vB_Cib_ssDNA_P1 supplies a new model system for studying the interactions between viruses and photoheterotrophs in the ocean.

So far, isolates of *Microviridae* are strictly lytic (Fane et al., 2006; Holmfeldt et al., 2013); however, *Microviridae*-related prophages have been found in genomes within the order *Bacteroidetes* (Krupovic and Forterre, 2011). Here, we present a ssDNA virus (vB_Cib_ssDNA_P1) that infects a marine alphaproteobacterium, and its closely related prophage in *N. tardaugens* NBRC16725, the first prophage found in an alphaproteobacterium. Collectively, these viruses define a new lineage of ssDNA viruses, the *Amoyvirinae*, which infect an evolutionary and ecologically important group of bacteria. The significance of these viruses in nature remains to be elucidated.

## Author Contributions

QZ, CS, and NJ conceived and designed the experiments. QC and YX conducted the experiments. QZ and QC analyzed the data. All of the authors assisted in writing the manuscript, discussed the results, and commented on the manuscript.

## Conflict of Interest Statement

The authors declare that the research was conducted in the absence of any commercial or financial relationships that could be construed as a potential conflict of interest.

## Footnotes

1 http://www.ncbi.nlm.nih.gov/gorf/gorf.html

## Supplementary Material

FIGURE S1 SDS–PAGE of vB_Cib_ssDNA_P1 proteins. Lane M, molecular weight standards; Lane CQ, vB_Cib_ssDNA_P1.

FIGURE S2 Unrooted maximum-likelihood phylogenetic tree based on the inferred amino-acid sequences of full-length replication initiator in *Microviridae* genomes. Bootstrap percentages from 100-replicate maximum-likelihood trees are shown. The scale bar represents a distance of 0.2 substitutions per site.
