# Supplementary figures and images for: A Virus Infecting Marine Photoheterotrophic Alphaproteobacteria (Citromicrobium spp.) Defines a New Lineage of ssDNA Viruses
Source: Front Microbiol. 2018 Jun 27;9:1418. doi: 10.3389/fmicb.2018.01418 (PMC6030365; doi:10.3389/fmicb.2018.01418)

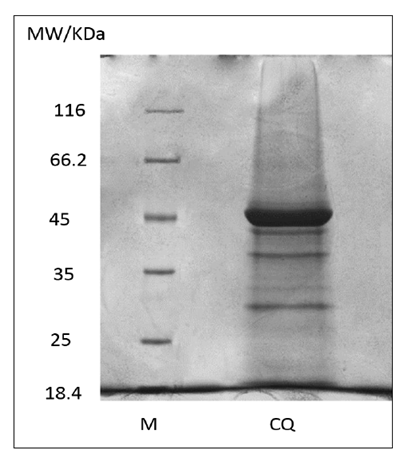

Supplement: FIGURE S1 — SDS–PAGE of vB_Cib_ssDNA_P1 proteins. Lane M, molecular weight standards; Lane CQ, vB_Cib_ssDNA_P1. [file Image_1.TIF]

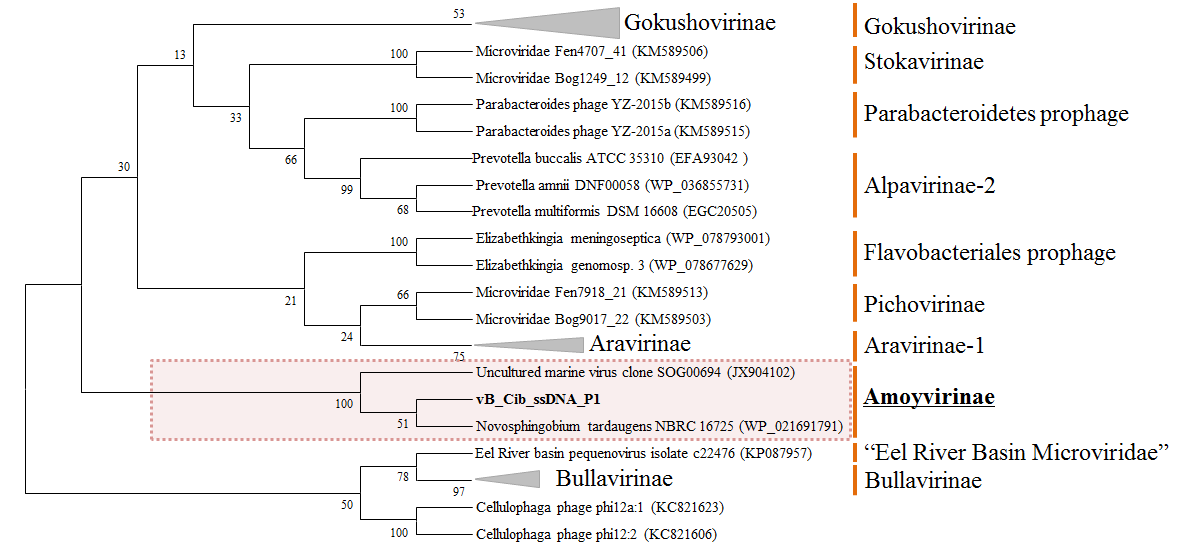

Supplement: FIGURE S2 — Unrooted maximum-likelihood phylogenetic tree based on the inferred amino-acid sequences of full-length replication initiator in Microviridae genomes. Bootstrap percentages from 100-replicate maximum-likelihood trees are shown. The scale bar represents a distance of 0.2 substitutions per site. [file Image_2.TIF]
